# Implementation and effectiveness of an intervention to Prevent and Reduce Involuntary Treatment at Home (PRITAH) in people living with dementia: protocol for a hybrid design type 3 quasi-experimental study

**DOI:** 10.1186/s12877-025-06508-1

**Published:** 2025-10-24

**Authors:** Klarissa Ponstein, Petra M.G. Erkens, Gerard J.P. van Breukelen, Jan P.H. Hamers, Michel H.C. Bleijlevens

**Affiliations:** 1 Department of Health Services Research, Care and Public Health Research Institute (CAPHRI), Faculty of Health, Medicine and Life Sciences (FHML), Maastricht University, Maastricht, 616, 6200 MD the Netherlands; 2 Living Lab in Ageing and Long-term Care, Maastricht University, Maastricht, the Netherlands; 3 Department of Methodology & Statistics, Care and Public Health Research Institute (CAPHRI), Faculty of Health, Medicine and Life Sciences (FHML), Faculty of Psychology and Neuroscience, Maastricht University, Maastricht, the Netherlands; 4 MeanderGroep Zuid-Limburg, Landgraaf, the Netherlands

**Keywords:** Implementation science, Hybrid design type-3 Effectiveness-Implementation study, Involuntary treatment, Dementia care, Long-Term care, Quality of care

## Abstract

**Background:**

Involuntary treatment refers to care where persons living with dementia are excluded from decision-making or do not provide consent. Despite serious consequences, involuntary treatment is often used in people with dementia receiving homecare. To address this, the Prevention and Reduction of Involuntary Treatment at Home (PRITAH) intervention was developed. A previous study indicated potential for scaling up PRITAH in professional homecare settings. However, implementing healthcare interventions like PRITAH is complex and often faces challenges. The current protocol describes a study that aims to: (1) gain a comprehensive understanding of the PRITAH- implementation in professional homecare settings, and (2) evaluate its effect on involuntary treatment use on people living with dementia at home.

**Methods:**

This quasi-experimental Hybrid Design Type-3 effectiveness-implementation study includes 88 case managers in the field of dementia care (CMDs) from four professional homecare organizations in Southern Limburg, the Netherlands. CMDs are divided into an intervention and control group stratified by geographical location. The intervention group receives the PRITAH-intervention, while the control group continues usual care. Data collection occurs at baseline (T0), 8 weeks (T1), and 20 weeks (T2) using questionnaires with open-ended and closed (Likert-scale) questions. Primary implementation outcomes include adoption, acceptability, appropriateness, feasibility, fidelity, and sustainability. Additionally, qualitative data from focus groups will be analysed. The primary effectiveness outcome is involuntary treatment use in people living with dementia at home, assessed via self-administered questionnaires completed by CMDs regarding ten randomly assigned clients within their caseload. Outcomes on CMD level include attitude, subjective norms, perceived behavioural control, and intention, which are prerequisites for behavioural change. Both descriptive analysis (quantitative) and content analysis (qualitative) evaluate implementation outcomes, while mixed (multilevel) linear regression models assess the effect of the PRITAH-intervention on involuntary treatment use.

**Discussion:**

This study provides insight into both implementation and effectiveness of the PRITAH-intervention in professional homecare settings. Due to potential contamination from communication among CMDs within the same organization and region, strict randomization is not feasible. Instead, a quasi-experimental design ensures a controlled comparison while maintaining real-world applicability. Including all available CMDs in the region enhances study’s validity, strengthening the intervention’s potential for broader implementation.

## Background

People living with dementia (PLWD) suffer from severe impairments in memory, reasoning, language, and ability to perform everyday tasks [1]. Despite progressive cognitive decline, most (70%) people living with dementia (PLWD) wish to continue living in their own home environment for as long as possible [2]. Many countries support this preference through aging-in-place policies [3–6]. Aging in place fosters independence and autonomy and encourages PLWD to rely on family and friends for support [7–9]. However, caring for PLWD can be demanding due to complex needs and many PLWD requires extensive family, social, and professional support [9–12]. As dementia progresses, the caregiving role may place significant physical, emotional, psychological, and financial burdens [10, 13, 14]. Additionally, since PLWD lose decision-making capacity, caregivers often make decisions on their behalf. This may lead to ethical dilemmas between ensuring safety and respecting personal freedom [15, 16]. When the PLWD is not involved in the decision-making process, does not consent to their care, or resist care provided by professional and informal caregivers, it is referred to as involuntary treatment [17, 18]. Involuntary treatment includes (a) non-consensual care, which is defined as “any type of care that limits the organization of a person’s own life”, such as forced ingestion of food or fluids and enforced personal hygiene [17, 19]; (b) physical restraints defined as “any action or procedure that restricts a person’s free body movement to a position of choice and/or prevents normal access to his/her body by any method attached or adjacent to a person’s body that he/she cannot control or remove easily [20]”; and (c) the use of psychotropic medication without consent, including off-label prescriptions (i.e. prescribing a drug outside its licensed indication or medication prescribed for an unapproved indication, such as managing challenging behaviour and dementia-related symptoms) [21].

Involuntary treatment negatively affects PLWD and their (informal) caregivers, increasing aggression, agitation, and injury risks. In turn, these consequences significantly diminish the overall physical and psychological well-being [22–24]. Despite these adverse effects, research indicates that involuntary treatment is still commonly used. In the Netherlands and Belgium, over half of the PLWD receiving professional homecare experience some type of involuntary treatment, with non-consensual care being the most common [17]. Involuntary treatment is used by informal caregivers in 73.6% of the cases, by nurses in 57.9% of the cases, and by general practitioners in 13.6% of the cases [25]. Caregivers often view involuntary treatment as a necessary component of their care, especially when safety or well-being is at risk. For instance, locking the front door to prevent a person with dementia from wandering outside. However, this mindset contributes to the continued use of involuntary treatment [18].

To reduce involuntary treatment use, the Netherlands has implemented legislation, called the Care and Coercion Act, which mandates that involuntary treatment should only be applied as an *ultimum remedium* (i.e. last resort) after exploring alternatives. The law requires long-term care organizations to revise policies on involuntary treatment and monitor the results [26]. In response, the Dutch Association of Nurses and Caregivers (V&VN) developed a practical guideline to help healthcare organizations comply with the law, which was published in 2024 [27]. Despite these regulatory efforts, the high prevalence of involuntary treatment in PLWD at home highlights the need to support caregivers in reducing involuntary treatment.

The Living Lab in Aging and Long-Term Care Limburg (Maastricht University) developed the intervention ‘Prevention and Reduction of Involuntary Treatment At Home (PRITAH)’ in 2018. PRITAH aims to support both formal and informal caregivers with knowledge and tools for preventing and reducing involuntary treatment use at home [28]. The PRITAH-intervention was based on the effective EXBELT intervention, which was designed to prevent and reduce physical restraint in nursing homes [29, 30]. A previously conducted exploratory mixed-methods study tested PRITAH’s feasibility in professional homecare organizations, showing potential for implementation after minor modifications. The study evaluated PRITAH at two time points, showing a positive impact on healthcare professionals’ subjective norms (T0 = 3.1 vs. T1 = 3.5 on a 5-point Likert scale) and perceived behavioural control (T0 = 3.6 vs. T1 = 3.9 on a 5-point Likert scale), which are prerequisites for behavioural change for preventing and reducing involuntary treatment use [28, 31]. These findings provide promising expectations for upscaling the PRITAH- intervention in professional homecare settings to reduce involuntary treatment use.

Implementing and upscaling healthcare interventions like PRITAH on a large scale is complex and often hindered with barriers that delay the implementation [32–34]. To address these challenges and ensure that evidence-based interventions like PRITAH reach their full potential, such as preserving the freedom and autonomy of PLWD, it is essential to gain more understanding of the implementation. However, focusing solely on implementation is insufficient, as the intervention’s effectiveness must also be ensured. By adopting a dual focus on both implementation and effectiveness, research can increase the likelihood of successful implementation, streamline the process, and save valuable time. Curran and colleagues introduced hybrid effectiveness-implementation designs to address both the effectiveness of an intervention and its practical implementation [35].

The current study focusses on the implementation of the PRITAH-intervention. Hence, the current study protocol describes a Hybrid Type 3 effectiveness-implementation study, as outlined by Curran et al. [35]. The aim of this paper is to provide a detailed protocol of our Hybrid Type-3 effectiveness-implementation study to (1) gain a comprehensive understanding of the PRITAH- implementation outcomes in professional homecare settings, and (2) evaluate its effect on involuntary treatment use on PLWD at home.

## Method

### Study design

This protocol outlines a quasi-experimental Hybrid Design Type-3 effectiveness implementation study.

### PRITAH- intervention

The Prevention and Reduction of Involuntary Treatment At Home (PRITAH) intervention aims to support healthcare professionals and informal caregivers with knowledge and tools that can help prevent and reduce involuntary treatment at home. The intervention consists of four key elements: (1) implementation of policy in homecare organizations aimed to discourage the use of involuntary treatment; (2) a plenary kick-off meeting and three workshops for CMDs including practical assignments and case studies; (3) on-the-job coaching and consultation by a specially trained case manager in the field of dementia or common care nurse; and (4) availability and utilization of alternative interventions for involuntary treatment alternatives to prevent involuntary treatment use [28]. A summary of the four key elements is presented in Table 1.

**Table 1:** Key elements of the PRITAH- intervention

Policy	The policy emphasizes that the organization will no longer apply involuntary treatment. It also sets clear conditions, such as raising awareness, adopting a multidisciplinary approach, maintaining communication with caregivers, and using alternatives. The policy is written and introduced to care professionals through the organization’s management.
***Education (2.5 h per workshop)***	Kick-off meeting - A general introduction to the PRITAH intervention and an overview of the three PRITAH- workshops by the research team. Workshop 1: increasing knowledge and awareness - Dilemmas about involuntary treatment, explaining the home care organization’s policy on its use, and providing an overview of current literature on its prevalence, risk factors, and consequences. Workshop 2: methodological approaches & alternatives - Methodological approach for situations involving involuntary treatment and exploring alternatives to prevent or reduce its use. Workshop 3: communication with stakeholders - Communication between caregivers, informal caregivers, and PLWD about involuntary treatment use, and using case studies to practice communication and for finding possible alternatives to involuntary treatment. Practical assignments between each workshop All workshops are given by specially trained nurses or case managers in the field of dementia care.
***Consultation and on-the-job coaching***	Consultation - Offering 30-minute consultation sessions, such as during team meetings, and providing guidance on preventing involuntary treatment in daily practice. On-the-job coaching - Available for questions and coaching in daily practice, with the coach serving as the primary point of contact for inquiries. *The trainers of the workshops will serve as a coach. *
***Alternatives***	Alternatives for involuntary treatment use will be discussed during the workshops and consultation sessions. A comprehensive list with alternatives will be provided to the participants. *The organizations are responsible for the availability of alternatives.*

### Study setting and sample

#### Setting

In the Netherlands, when informal caregivers are no longer able to care for PLWD or meet their needs or wishes, professional support from general practitioners and homecare is provided. Homecare organizations in the Netherlands are organized in small districts, each managed by a team of nurses. Within each district team, a district nurse is responsible for planning, coordinating, delivering, and assessing care within a PLWD home environment [36]. Depending on the support needed by the PLWD, other members of the district team may include common care nurses, vocationally trained nurses, nursing assistants, and domestic workers. Additional to usual homecare practices, CMDs play an important role in the care for PLWD at home. They are specially trained nurses or social workers involved in the care process once a diagnosis of dementia has been made. Their primary role is to act as a bridge between professionals and organizations, streamlining care delivery for PLWD and enhancing communication among stakeholders. CMDs are often perceived as key sources of support and guidance, remaining closely involved with PLWD and their caregivers from diagnosis through to institutionalization or end of life [37].

#### Sample

**Fig. 1:**
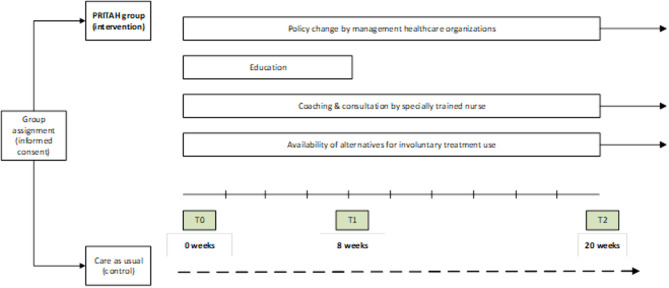
Study-design

The study will take place within the region of South Limburg in the Netherlands, home to a population of 402,700 individuals [38]. Of this population, 27.4% are 65 years or older and 2.2% (8,660 individuals) are diagnosed with dementia [39, 40]. In 2020, CMDs supported approximately 33–41% (i.e. 2,858 to 3,551) of the people living with dementia at home in the region [41]. In this large region, 88 CMDs employed by four professional homecare organizations are responsible for the dementia care in this region. Each CMD manages a caseload of approximately 55 clients (i.e. PLWD) in a fulltime position or proportionally in a part time position. All 88 CMDs working in the region of South Limburg in the Netherlands are invited to participate in the study on the implementation of the PRITAH-intervention. The CMDs are employed across four organizations in two regions and are each assigned to a specific city district or village within that region. To avoid contamination, all CMDs within the same district or village are grouped together, resulting in multiple clusters. These clusters are then allocated to either the intervention or the control condition, ensuring that CMDs within a given cluster did not interact across conditions. The CMDs in the intervention group receive training in batches of 10 to 14 people, with a two-week delay between the start of each batch’s training. CMDs in the control group will provide care as usual, continuing their routine work. They will not receive any PRITAH components (policy, kick-off meeting and training, on-the-job coaching and consultation, and alternatives for involuntary treatment). Moreover, CMDs in the control group are free to address involuntary treatment according to their usual practice in their respective organization. CMDs on maternity leave, long-term sick leave, nearing retirement, or newly recruited during the PRITAH-implementation period are excluded from the study. In a nested design, as part of the effect study, all 88 participating CMDs will report on the use of involuntary treatment by formal and informal caregivers for 10 randomly selected clients within their caseload to provide insights at the client level. If a client drops out of the study due to discontinuation of care (i.e. institutionalization or end of life), they will be replaced by another randomly assigned client from the caseload of the CMD. Therefore, we anticipate collecting 880 responses on client level. See Fig. 1.

As all available CMDs from the participating care organizations will be invited to participate in the study, a formal sample size calculation will not be performed. A separate sample size calculation for clients is unnecessary. The statistical power of the study primarily depends on the number of CMDs, as the variation in intervention status (yes/no) occurs between CMDs rather than between clients within the same CMD [42–44].

### Measurements

In the study, we will determine whether PRITAH is well implemented within these organizations. In parallel, the study will also measure the effect of the PRITAH-intervention by examining the types of involuntary treatment used in PLWD at home. Questionnaires will be collected at three points in time. A baseline measurement (T0) will be done after the kick-off meeting, but before the first PRITAH- workshop. T1 will be measured directly after the third PRITAH-workshop and T2 will be measured 12 weeks later (see Fig. 1). Both the control group and the intervention group will be measured at the same time intervals. In addition, data from observations from the PRITAH-training and evaluative focus group meetings will be collected during and after the PRITAH-training period.

#### Implementation outcomes

Primary outcomes for the PRITAH-implementation include adoption, acceptability, appropriateness, feasibility, fidelity, and sustainability, as defined by Proctor [45–47]. These outcomes are used to capture the initial uptake, practical integration, and long-term embedding of PRITAH within the healthcare organizations and CMDs [48]. The outcomes will be assessed using self-administered questionnaires based on Proctor’s definitions, PRITAH-training observations, PRITAH-training evaluation forms, attendance lists, and focus groups with PRITAH-trainers and managers of the organizations [46]. The questionnaires contain multiple statements for each outcome measure, with responses rated on a 5-point Likert scale ranging from totally disagree to totally agree. The development of the questionnaire is based on the Acceptability of Intervention Measure (AIM), Intervention Appropriateness Measure (IAM), and Feasibility of Intervention Measure (FIM) [45]. Furthermore, both the phrasing and the content of the statements were reviewed by experienced researchers in implementation science to assure face validity. To complement data on the implementation outcomes, additional qualitative data will be collected. During the nine PRITAH-workshop sessions (three training groups with three sessions each), a member of the research team takes observations through field notes to assess whether the trainers adhered to the protocol and whether modifications were made. PRITAH-training evaluation forms, containing both closed and open-ended questions are distributed and completed after each workshop to gather feedback on that specific session. The output of these evaluation forms will be used as input for the two focus group meetings with both PRITAH-trainers and managers from the participating organizations. Lastly, attendance lists are recorded. See Table 2 for a summary of the implementation outcomes and measurements.

**Table 2:** Implementation outcomes

Implementation outcome (Proctor, 2011)	Operationalisation description	Measurement	Target group	Timepoint
Adoption	The intention and decision to engage with PRITAH and its four key elements, reflecting how well the intervention is embraced by healthcare professionals	Questionnaire PRITAH-training evaluation forms Focus group meeting	CMD (IG) Trainers Managers	T0, T1
Acceptability	The perception among CMDs that PRITAH is agreeable, acceptable, and satisfactory, measuring how well the intervention is received and appreciated by those who are expected to implement it	Questionnaire PRITAH-training evaluation forms	CMD (IG)	T0, T1
Appropriateness	The perceived fit, relevance, and compatibility of the PRITAH- intervention within the homecare setting and within the needs of CMDs and PLWD, evaluating whether the intervention is seen as suitable for the context (i.e. professional homecare organizations) in which it is being applied	Questionnaire	CMD (IG)	T0, T1
Feasibility	The practicality of implementing PRITAH within the organization, evaluating its fit and functionality in the professional homecare settings	Questionnaire PRITAH-training evaluation forms Focus group meeting	CMD (IG) Trainers Managers	T1, T2
Fidelity	The degree to which PRITAH-intervention is delivered as intended, ensuring that the intervention is implemented accurately and consistently according to the designed protocol and guidelines	Observations (field notes) Attendance lists	Members from the research team Trainers	T1
Sustainability	How well the PRITAH- intervention is integrated into the organization’s routine processes and embedded into its culture through established policies and continuous practices	Questionnaire	CMD (IG)	T2

*T0* = 0 weeks (before PRITAH-workshop 1), *T1* = 8 weeks after start PRITAH-workshop 1 (directly after finishing PRITAH-workshops), *T2* = 20 weeks after start PRITAH-workshop 1 (12 weeks after finishing PRITAH-workshops

*IG* Intervention Group

#### Effect outcomes

The primary outcome of the PRITAH- effectiveness is the use of involuntary treatment in PLWD at home, including non-consensual care, physical restraints, and psychotropic medication (i.e. off-label use). Involuntary treatment use will be measured at baseline (T0), T1, and T2 using questionnaires spread under CMD’s. The questionnaire is based on the questionnaire that was developed by Hamers et al., which was also used to assess the prevalence of involuntary treatment by Moermans et al. [17, 19]. It comprises 25 types of involuntary treatment categorized into the two forms (12 types of physical restraint, 13 types of non-consensual care), and includes two separate questions about the use of psychotropic medication. CMDs will use the self-administered questionnaire to indicate whether each type of involuntary treatment is used for ten randomly assigned PLWD within their caseload. Secondary outcome measures involve mechanisms of impact assessed through a self-administered questionnaire based on the Theory of Planned Behaviour, which was used by Mengelers in the pilot/feasibility study for PRITAH-intervention [31, 49]. CMDs’ attitudes (20 items), subjective norms (5 items), perceived behavioural control (9 items), and intention (4 items) will be rated on a 5-point scale ranging from 0 (totally disagree) to 5 (totally agree). Four additional items are included to gather information about the CMDs characteristics, including age, educational background, work experience, and workload perception. Lastly, three additional questions address whether the CMD discusses involuntary treatment use with the person with dementia, and/or their formal and informal caregivers. See Table 3 for an overview.

**Table 3:** Effect outcomes

Effect outcomes	Description	Measurement	Target group	Timepoint
Involuntary treatment use in PLWD at home	The extent to which involuntary treatments (i.e. non-consensual care, physical restraints, and psychotropic medication) are used in PLWD at home	Questionnaire	CMD (IG & CG)	T0, T1, T2
Mechanisms of impact:	Mechanisms of impact regarding involuntary treatment use based on the Theory of Planned Behaviour (49)	Questionnaire	CMD (IG & CG)	T0, T1, T2
*Attitude*	Settled way of thinking or feeling about involuntary treatment use			
*Subjective norms*	Perception of social pressure from significant others regarding involuntary treatment use			
*Perceived behavioural control*	Sense of ease or difficulty in performing behaviours related to preventing involuntary treatment use			
*Intention*	Readiness or commitment to engage in behaviours related to preventing involuntary treatment use			
Discussions about involuntary treatment use	Whether the CMD discusses involuntary treatment use with the PLWD, formal caregivers (e.g., healthcare staff), and/or informal caregivers (e.g., family)	Questionnaire	CMD (IG & CG)	T0, T1, T2

T0 = 0 weeks (before PRITAH-workshop 1); T1 = 8 weeks after start PRITAH-workshop 1 (directly after finishing PRITAH-workshops); T2 = 20 weeks after start PRITAH-workshop 1 (12 weeks after finishing PRITAH-workshops)

IG = Intervention group; CG = Control group

### Data analyses

Depending on the distribution of the data means with standard deviations or medians with interquartile range will be presented for interval and ratio variables. For ordinal and nominal variables frequencies and percentages will be reported. Data will be analysed using SPSS statistics version 28. Cases will be excluded for whom no data have been collected at any of the three timepoints. Qualitative data will be analysed by using qualitative analysis software MAXQDA 24.

#### Implementation outcomes

##### Quantitative data

Descriptive statistics, including frequencies, means, and standard deviations for each item, will be used to summarize the CMDs’ responses to the self-administered questionnaires, providing an overview of the levels of adoption, acceptability, appropriateness, fidelity, feasibility, and sustainability. To complement this data, frequencies and percentages will be reported for the closed-ended questions in the PRITAH-evaluation forms.

##### Qualitative data

Data from the open-ended questions in the PRITAH-training evaluation forms will serve as input for the focus groups with the PRITAH-trainers and mangers of the organizations. To facilitate the analyses, the responses to the open-ended questions will be uploaded to the qualitative analysis software MAXQDA [50]. Analysis will be done through an iterative process involving multiple rounds of analysis and evaluation. A conventional content analysis approach will be used to explore the experiences and perceptions of the CMDs, trainers, and managers regarding the PRITAH-training and implementation [51, 52] This inductive approach avoids the use of predetermined categories, allowing the data to guide the categories and names for categories. The researcher will begin by familiarizing themselves with the data, reading all transcripts multiple times to obtain a sense of the whole. Subsequently, a line-by-line analysis will be performed to identify codes that capture key concepts. The researcher then notes initial impressions and thoughts. As this process continues, labels for codes emerge to form the initial coding scheme. These codes are then grouped into (sub)categories based on their relationships, creating meaningful clusters. This categorization process will be reviewed and refined in collaboration with the co-authors to ensure consistency and accuracy.

Data from the focus group meetings will be transcribed using an automatic speech recognition model (ASR), assisted by a research assistant [53]. The same analytical approach as for the open-ended questions will be used for this data. Since the findings from the open-ended questions serve as input for the focus groups, similar categories are likely to emerge. Although potential overlap, we will distinguish between two perspectives in which the questionnaires reflect the perspectives of CMDs, and the focus groups capture the perspectives of trainers and managers. As this remains an inductive process, the research team will be open to the emergence of new categories.

To assure trustworthiness, credibility is established through investigator triangulation, as two researchers will independently code the data from the open-ended questions and the focus groups. Additionally, the output from the open-ended questions serve as input for the focus groups, which can also be seen as a form of member checking [54].

##### Effect outcomes

Outcomes at the CMD level (attitude, subjective norms, perceived behavioural control, and intention) will be analysed with mixed (multilevel) regression, with linear regression for continuous outcomes and generalized linear regression (e.g., logistic, ordinal, gamma) for categorical outcomes. A total score per CMD or client per time point will be calculated for each outcome measure if multiple items measure the same construct and share the same response format (e.g. all items are binary or all items use a 5-point Likert scale). Repeated outcome measures are nested within CMDs. The fixed (predictor) part of the mixed model will include intervention (yes/no), region, care organization, and time point (t0, t1, t2; week 0/8/20), along with age, gender, educational level, work experience in care for older people, work experience in professional home care, current position, number of working hours, and hours working with individuals with dementia. Additionally, interaction of each predictor with time will be evaluated. The random part of the model will account for unexplained outcome variation between CMDs, which is allowed to change over time, by assuming an unstructured residual covariance matrix (marginal model). Predictor by time interactions will be deleted if not significant, with exception of the key interaction between intervention and time. Giving the nonrandomized treatment assignment, additional analysis will be (a) comparing treated and controls with respect to the baseline outcome measurement, adjusted for all abovementioned confounders, and (b) repeating the above mixed regression, but now with the baseline outcome as covariate instead of as repeated measures. This approach will help assess Lord’s paradox as a sensitivity/robustness analysis [43, 55].

All participating CMDs will be included into the analyses with all available data without imputing missing outcome measurements using maximum likelihood estimation. This approach is valid under the Missing At Random (MAR) assumption and is asymptotically equivalent to multiple imputation when using an appropriate imputation model [56]. Additionally, outcome missingness will be analyzed as a separate dependent variable using logistic regression (missingness at any given time point: yes/no) or mixed logistic regression for repeated measures (missingness at each time point), depending on the frequency and pattern of missing data. Covariates that predict missingness will be identified and incorporated into the primary outcome analyses.

Outcomes at the client level ((total score on the involuntary treatment questionnaire, and score per subscale (i.e., physical constraint, non-consensual care, the use of psychotropic medication)) will be analyzed in the same way as outcomes at the CMD level, with two modifications. First, predictors at the client level such as the client’s age, gender, living situation, informal caregiver, diagnosis, and presence of professional of homecare will be included as covariates. Second, unexplained outcome variation at the client as well as at the CMD level will be modeled with an unstructured covariance matrix [42].

### Ethical considerations

The Medical Ethical Committee of Zuyderland Medical Centre (METCZ20230080) determined on February 10th 2025 that this study was not subject to the Medical Research Involving Human Subjects Act. The PRITAH intervention itself and the small-scale implementation (feasibility and applicability) study was approved by the Medical Ethical Committee of Zuyderland Medical Centre (METC20180101) on August 30th 2018.

All participants in the study will receive an information letter to inform them about the aims and expectations of participating in the PRITAH- implementation study and will be asked to give their informed consent.

## Discussion

The current protocol describes the design of a quasi-experimental Hybrid Design Type-3 effectiveness-implementation study. Implementing new healthcare interventions into real-world practices can be challenging and time consuming [57]. Challenges regarding implementation often depends on the context in which the intervention is implemented, the individuals in the process, and the characteristics of the intervention itself [58, 59]. To address these challenges, conducting a context analysis is essential [58, 60]. Therefore, the current protocol was developed based on the results from a prior context analysis for the PRITAH-implementation (*to be submitted*). In two focus groups with policy staff members, a district nurse, CMDs, managers, and directors, we explored barriers and facilitators using the Consolidated Framework for Implementation Research (CFIR) [58, 60]. Overall, the context analysis revealed that both participating home care organizations support the implementation of PRITAH to prevent or reduce involuntary treatment at home.

Although randomized controlled trials (RCTs) are considered the gold standard in research, our study uses a quasi-experimental design. This type of design is more often recognized for their use in implementation studies where strict randomization may be infeasible or unfitting [61]. In our study, communication between CMDs within the same care organisation and region might induce treatment contamination. Hence, stratification based on region and district is necessary.

A Hybrid Type 3 Design is well suited for this study as the success of the PRITAH-intervention is determined both by how well it can be implemented into existing homecare settings and by its effect on client outcomes. Recommended conditions for a Type 3 Design, as outlined by Curran and colleagues, further support his approach The first condition is that, although the full effectiveness of the PRITAH intervention has yet to be established, research has demonstrated sufficient indirect evidence of its effectiveness, warranting further exploration of its implementation. Another supportive condition for this type of design is the legislation on involuntary treatment (Care and Coercion Act) and the in 2024 released guidelines, which creates a good momentum for implementation [35, 62].

The reliance on self-reported measures to assess both implementation and effect outcomes is a potential limitation. Self-reported data may be susceptible to different types of information biases, which could affect the reliability of the findings [63]. However, it should be emphasized that the target group of the current study involves PLWD living at home (i.e. behind closed doors). As clients are spread across individual households rather than concentrated in one setting, we depend on those who visit them. Therefore, self-reported data from CMDs represent the most feasible and informative approach to assess the outcomes. Importantly, while these data are reported by the CMDs participating in the PRITAH study, they concern the use of involuntary treatment by both formal and informal caregivers who are responsible for care in the client’s home situation. This provides a broader picture of involuntary treatment use beyond the CMD’s own practice. In addition, self-reporting encourages CMDs to reflect on their own practices and increases awareness of involuntary treatment use. Lastly, this approach is necessary given the logistical and time-consuming restraints of independent measurement (e.g. document analyses, interviews, observations), which might lead to the exclusion of participants as well as lower response rates.

We consider as a strength of this study the choice of CMDs as the study sample. CMDs play an important role in the care for PLWD at home by acting as a bridge between formal and informal care. Through their close collaboration with district teams, they are well positioned to disseminate the principles of the PRITAH-intervention and integrate them into daily practices. This central role makes CMDs key stakeholders, not only during the implementation but also in achieving the intervention’s intended effect on reducing involuntary treatment use in PLWD at home. Another strength of the study is the inclusive approach to ensure participation of all 88 CMDs working for four professional homecare organizations who meet the inclusion criteria. Therefore, within the study a broad range of experiences and practices is captured given the fact that each CMD has a large caseload. The diversity makes sure that the findings are more reflective of real-world settings, which in turn increases generalizability. Lastly, including all CMDs provides a more holistic view of the experiences towards the implementation, which is necessary for addressing barriers in future implementation efforts.

## Conclusion

Based on existing literature, previous research, and a context analysis this study aims to explore how to successfully implement the PRITAH-intervention and assess its effectiveness in preventing and reducing involuntary treatment. By using a quasi-experimental design and incorporating insights from a broad context analysis, we addressed the challenges of real-world implementation in this protocol. This protocol may serve as an example for the implementation of other complex interventions in long-term care settings for older people, offering a systematic and reproducible approach.

## Data Availability

No datasets were generated or analysed during the current study.

## Abbreviations

PRITAH: Prevention and Reduction of Involuntary Treatment At Home
CMD: Case Manager in the field of Dementia care
PLWD: People Living with Dementia
V&VN: Dutch Association of Nurses and Caregivers
AIM: Acceptability of Intervention Measure
IAM: Intervention Appropriateness Measure
FIM: Feasibility of Intervention Measure
ASR: Automatic Speech Recognition model
